# Using Chat Generative Pre-Trained Transformer for teaching and learning: A survey among health professions educators

**DOI:** 10.4102/jcmsa.v4i1.442

**Published:** 2026-08-10

**Authors:** Niren R. Maharaj, Christolene M.B. Saaiman, Dirk T. Hagemeister

**Affiliations:** 1 Department of Obstetrics and Gynaecology, School of Clinical Medicine, Faculty of Health Sciences, University of the Free State, Bloemfontein, South Africa; 2 Office of the Dean, Division of Health Sciences Education, Faculty of Health Sciences, University of the Free State, Bloemfontein, South Africa; 3 Department of Family Medicine, School of Clinical Medicine, Faculty of Health Sciences, University of the Free State, Bloemfontein, South Africa

**Keywords:** ChatGPT, health professions education, knowledge, attitudes, practices

## Abstract

**Background:**

Chat Generative Pre-Trained Transformer (ChatGPT) has the potential to transform Health Professions Education (HPE), but its integration into educational practice is likely to be influenced by the knowledge, attitudes and practices of health professions educators. The aim was to assess these educators’ knowledge and awareness, attitudes and practices regarding ChatGPT.

**Methods:**

A cross-sectional study was conducted at a large teaching hospital using a structured questionnaire. Data were collected and analysed quantitatively to compare responses across professional groups.

**Results:**

A convenience sample of 360 healthcare professionals was recruited, comprising medical practitioners (MPs) (46.4%), health and rehabilitation sciences practitioners (HRSP) (13.3%) and nursing practitioners (NP) (40.3%). Most participants (*n* = 233; 64.9%) were aged 31–50 years and self-identified as female (*n* = 242; 67.4%). Awareness of ChatGPT was higher among MPs (98.8%) and HRSPs (95.8%), compared with NPs (65.5%). More than half of the respondents (55.3%) agreed that ChatGPT improves teaching and learning and that students should use it (54.9%), while 81.9% supported its further development. Concerns included incorrect information (63.2%), plagiarism (78.0%) and reduced independent thinking (64.5%). The current use was low (34.9%) and only 20 (19.0%) had received prior training. Most respondents reported easy access (81.7%) and requested further training (92.5%).

**Conclusion:**

Differences in knowledge, attitudes and practices exist among health professionals and may influence the integration of ChatGPT into HPE. Further research and targeted artificial intelligence (AI) literacy programmes are recommended to support its implementation.

**Contribution:**

The study provided valuable insights that could contribute to the development of AI-inclusive curricula in HPE.

## Introduction

Chat Generative Pre-Trained Transformer (ChatGPT) is a generative artificial intelligence (AI) based large language model (LLM) that has become the fastest-growing internet application in history.^1^ It is currently applied in different fields, including in Health Professions Education (HPE), as it offers a variety of potential benefits such as patient–doctor simulation, practice in clinical decision making, interactive learning experiences, personalised feedback and adaptive learning tailored to individual learners’ needs.^2^ However, concerns such as copyright infringement, a lack of transparency and originality, plagiarism, inaccurate content and unethical practice are some of the challenges associated with its use.^3^ In resource-constrained settings, the cost-free public access to ChatGPT can be used to enhance student learning outcomes and increase access to information.^4^ The World Medical Association (WMA) has advocated for the inclusion of modern technology into HPE.^5^ However, guidelines on the use of AI tools such as ChatGPT in HPE should be evidence-based and pedagogically sound.^6^ Currently, there is a lack of conclusive evidence-based guidelines on the use of AI in HPE.

Moreover, existing research on ChatGPT originates predominantly from high-income countries (HICs), and study’s findings may not be generalisable globally.^2^ Limited data are available from low- and middle-income countries (LMICs), including South Africa, to inform the development of pedagogically sound evidence-based guidelines for the local context. Furthermore, the knowledge, attitudes and practices of health professions educators may influence their acceptance of ChatGPT for teaching and learning.^7,8^ However, these perspectives have not been evaluated adequately among health professionals in a teaching hospital setting where interprofessional education and work-based learning occur.

To address this gap, the aim of the study was to explore the knowledge, attitudes and practices of health professionals regarding ChatGPT for teaching and learning in a large interprofessional teaching hospital setting. The study is underpinned by the Technology Acceptance Model (TAM), a theoretical framework highlighting that the ‘perceived usefulness and ease of use’ influence individuals’ attitudes towards using new technology.^9^ The study provided rare and valuable insights that could contribute to the development of AI-inclusive curricula in HPE.

## Research methods and design

### Study design

The study employed a cross-sectional descriptive design, using a paper-based questionnaire as the data collection tool.

### Population

Participants were recruited from Universitas Academic Hospital, a central teaching hospital in Bloemfontein, South Africa, for voluntary participation. The participants were grouped as follows: MP (specialists and registrars), NP (nursing auxiliaries, enrolled and registered nurses, midwives, specialist nurses and nursing operational managers) and health and rehabilitation sciences practitioners (HRSPs) (physiotherapists, occupational and speech and hearing therapists, dieticians and radiographers). A final convenience sample of 360 participants comprising medical practitioners (MP) (*n* = 167), nursing practitioners (NP) (*n* = 145) and HRSP (*n* = 48) was obtained.

### Data collection

Participants’ attitudes towards ChatGPT were assessed using a paper-based, self-administered questionnaire. Six statements, reflecting either support for or concerns about ChatGPT were rated using a 3-point Likert scale analysed as 1 = agree, 2 = undecided and 3 = disagree. The statements were based on the existing literature and refined by the investigators to align with the study objectives. Although other domains, such as the use of LLMs in formative and summative assessment, are also important, these were beyond the scope of this study.

The statements included: (1) ChatGPT improves the standard of teaching and learning (educational value); (2) students should use it (technology adoption); (3) it should be developed further for use in HPE (educational innovation); (4) it may provide incorrect information (accuracy and reliability); (5) plagiarism is a concern (academic integrity and ethical concern); and (6) its potential to reduce the ability of students to think independently (cognitive concern).

The statements on practices were related to: (1) current use of ChatGPT for teaching; (2) training received; (3) accessibility in the institution; and (4) a need for further training. A binary scale was applied in this section of the questionnaire, with only yes and no provided as options.

Participants’ responses were captured using the research electronic data capture (REDCap) template (REDCap Consortium; Nashville, TN, USA) to facilitate analysis and ensure confidentiality. The data capture process was cross-checked by an independent research assistant.

### Pilot study

A pilot study was conducted with 10 participants to evaluate the feasibility of the questionnaire, clarity and comprehensibility, and no modifications were required. Data from these participants were not included in the final analysis. Cronbach’s alpha was not performed, as the questionnaire was developed specifically for this exploratory study rather than as a standardised psychometric instrument.

### Statistical analysis

Descriptive statistics were used to analyse the general characteristics of the study population and the questionnaire responses. Numerical variables were summarised by means and standard deviations, or medians and percentiles. Categorical variables were summarised by frequencies and percentages. Differences between groups were evaluated using Chi-square or Fisher’s exact test for unpaired data. The analysis was carried out by the Department of Biostatistics, using SAS version 9.4 (SAS Institute Inc.; Cary, NC, USA).

### Ethical considerations

Ethical approval was obtained from the Health Sciences Research Ethics Committee (HSREC) of the University of the Free State (Ethics approval number UFS-HSD2024/1838/2502), and permission was granted by the Free State Department of Health to conduct the study in a public health facility. Voluntary written informed consent was obtained prior to participation. The questionnaires were coded to ensure anonymity of the participants, and all data were kept confidential.

## Results

### Demographic characteristics

The group consisted of 167 (46.4%) MP, 145 (40.3%) NP and 48 (13.3%) HRSP. The demographic data section of the questionnaire was analysed for 359 participants, as this section was incomplete in one questionnaire. A statistically significant difference in the age distribution was observed across the professional groups (*p* < 0.0001). The majority of respondents (*n* = 233, 64.7%) were between 31 years and 50 years of age, aligning with the general age profile of employees at the institution and national data, which indicate that approximately 56.9% of public healthcare employees in South Africa are aged between 35 years and 54 years.^10^

There was a significant difference in the gender distribution (*p* < 0.0001) of respondents, with a higher proportion identifying as female (*n* = 242; 67.4%). This was broadly consistent with the institutional gender profile, where 76.4% of employees are female, and aligned with national data indicating that 78.1% of the public sector healthcare workforce in South Africa are female.^10^

Regarding educational qualifications, one third of respondents held a bachelor’s degree (*n* = 125; 34.8%), while a similar proportion (*n* = 119; 33.1%) had obtained postgraduate qualifications. The remaining respondents held diplomas and certificates. The minimum qualifications obtained by the respondents aligned with the requirements for professional registration in the different professional disciplines.

### Knowledge of Chat Generative Pre-Trained Transformer

**FIGURE 1:**
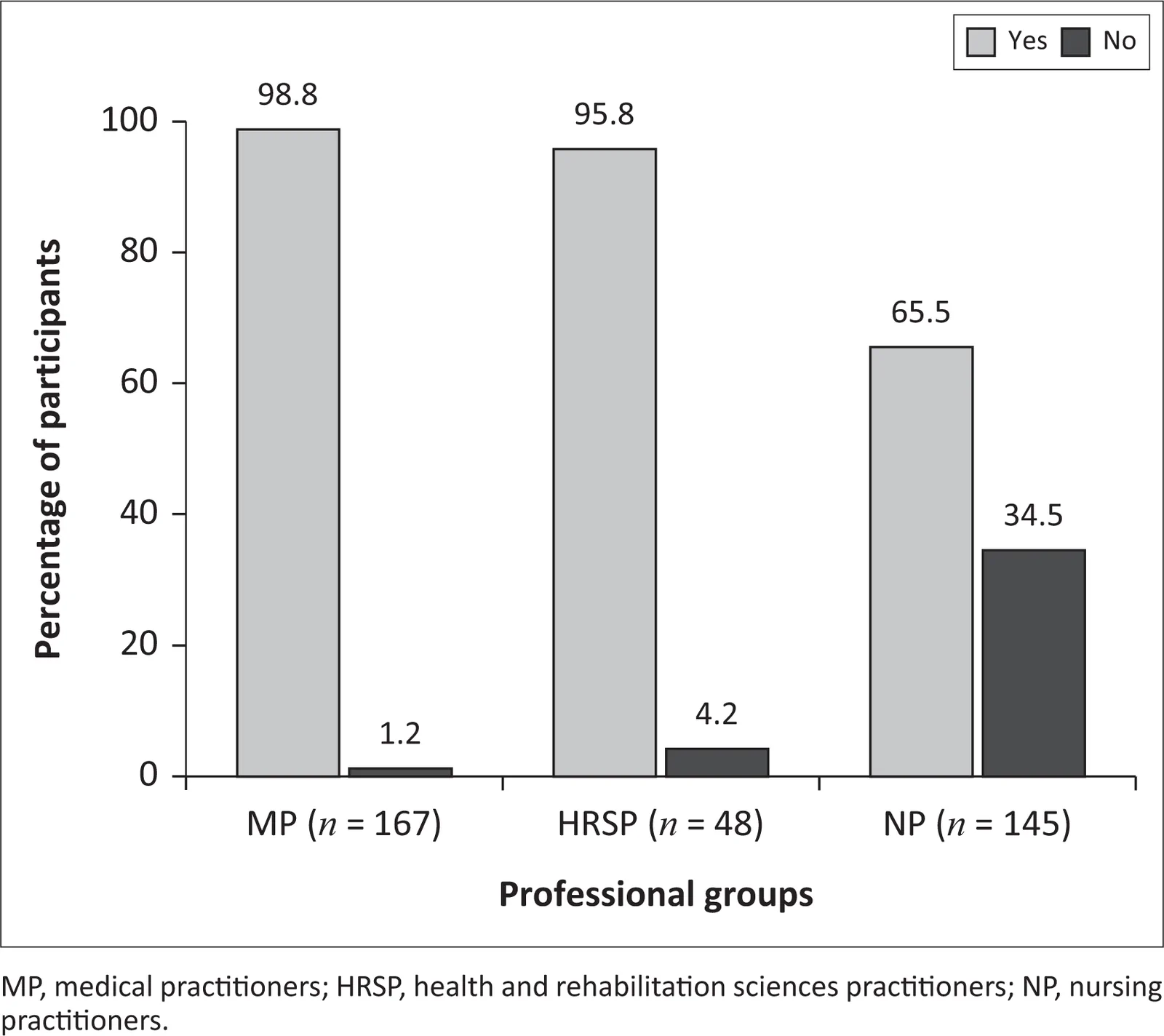
Distribution of participants in the different professional groups who had heard of ChatGPT.

Overall, the majority of all the study respondents (*n* = 306; 85.0%) reported that they had heard of ChatGPT, compared with a smaller proportion (*n* = 54; 15.0%) who had not heard of it. Variations occurred across the professional groups regarding knowledge and awareness of ChatGPT (Figure 1). More than 95% of respondents in the MP and HRSP groups had heard of ChatGPT, compared to 65.5% of respondents in the NP group.

### Attitudes

The responses to statements in support of ChatGPT are presented in Table 1. Of the 306 respondents who were aware of ChatGPT, 304 provided complete data for analysis, while two were excluded. Overall, slightly more than half of the respondents agreed that ChatGPT improves teaching and learning (*n* = 168; 55.3%) and that students should use it (*n* = 167; 54.9%). However, a substantial proportion of respondents (*n* = 249; 81.9%) agreed that the use of ChatGPT should be developed further for teaching and learning. The differences across groups were statistically significant for all the statements in support of ChatGPT.

**TABLE 1:** Participants’ responses to statements in support of and concerns about Chat Generative Pre-Trained Transformer.

Statements and responses	Group								*p*-value
	MP (*n* = 161)		HRSP (*n* = 46)		NP (*n* = 97)		Total (*n* = 304)		
	*n*	%	*n*	%	*n*	%	*n*	%	
**Support statements**									
(1) Improves teaching and learning	-	-	-	-	-	-	-	-	< 0.0001
Agree	67	41.6	17	36.9	84	86.6	168	55.3	-
Undecided	70	43.5	21	45.6	11	11.3	102	33.6	-
Disagree	24	14.9	8	17.4	2	2.0	34	11.2	-
(2) Students should use it	-	-	-	-	-	-	-	-	< 0.0001
Agree	70	43.5	23	50.0	74	76.3	167	54.9	-
Undecided	65	40.3	19	41.3	14	14.4	98	32.2	-
Disagree	26	16.1	4	8.7	9	9.3	39	12.8	-
(3) Should be developed	-	-	-	-	-	-	-	-	0.0079
Agree	122	75.8	37	80.4	90	92.8	249	81.9	-
Undecided	27	16.8	6	13.0	11	11.3	44	18.0	-
Disagree	8	4.9	3	6.5	0	0.0	11	3.7	-
**Concern statements**									
(4) Can give incorrect information	-	-	-	-	-	-	-	-	< 0.0001
Agree	119	73.4	29	63.0	44	45.4	192	63.2	-
Undecided	35	21.8	14	30.4	25	25.8	74	24.3	-
Disagree	7	4.3	3	6.52	28	28.9	38	12.5	-
(5) Plagiarism is a concern	-	-	-	-	-	-	-	-	0.0831
Agree	127	78.9	41	89.1	69	71.1	237	78.0	-
Undecided	26	16.1	2	4.3	18	18.5	46	15.1	-
Disagree	8	4.9	3	6.5	10	10.3	21	6.9	-
(6) Reduces independent thinking	-	-	-	-	-	-	-	-	0.0001
Agree	85	52.8	39	84.8	72	74.2	196	64.5	-
Undecided	43	26.7	5	10.9	11	11.3	59	19.4	-
Disagree	33	20.5	2	4.3	14	14.4	49	16.1	-

MP, medical practitioners; HRSP, health and rehabilitation sciences practitioners; NP, nursing practitioners.

Table 1 further summarises the responses to statements of concern about ChatGPT. The majority of respondents overall expressed concerns about the potential of ChatGPT to provide incorrect information (*n* = 192; 63.2%), foster plagiarism (*n* = 237; 78.0%) and reduce students’ capacity to think independently (*n* = 196; 64.5%). Although not significantly different across groups (*p* = 0.0831), plagiarism emerged as a dominant concern, being expressed by the largest proportion of respondents across the professional groups.

### Practices

The responses to practices related to ChatGPT use are presented in Table 2. The total number of responses varied from 104 to 304 across the four questions in this section. The current use of ChatGPT among the professional groups overall was low (*n* = 106; 34.9%), comprising just over a third of all the respondents. Only 19.0% (*n* = 20/105) of respondents reported receiving prior training on ChatGPT. A statistically significant difference (*p* = 0.0252) was observed between the professional groups with regard to training received on ChatGPT. Most of the respondents using ChatGPT (*n* = 85/104; 81.7%) agreed that the platform was easily accessible within the institution, and a substantial majority (*n* = 98/106; 92.5%) indicated that they would like to receive further training on its use and applications in the academic context.

**TABLE 2:** Participants’ responses to practices related to Chat Generative Pre-Trained Transformer use.

Statements and responses	Group								*p*-value
	MP (*n* = 161)		HRSP (*n* = 46)		NP (*n* = 97)		Total (*n* = 304)		
	*n*	%	*n*	%	*n*	%	*n*	%	
**(1) Currently using it for teaching**	-	-	-	-	-	-	-	-	0.0665
Yes	66	40.1	14	30.4	26	26.8	106/304	34.9	-
No	96	59.6	32	69.6	70	72.2	198/304	65.1	-
**(2) Received training on ChatGPT**	-	-	-	-	-	-	-	-	0.0252
Yes	8	4.9	3	6.5	9	9.3	20/105	19.0	-
No	58	36.0	11	23.9	16	16.5	85/105	81.0	-
**(3) Easily accessible in institution**	-	-	-	-	-	-	-	-	0.6152
Yes	52	32.3	13	28.3	20	20.6	85/104	81.7	-
No	13	8.1	1	2.2	5	5.2	19/104	18.3	-
**(4) Would like further training**	-	-	-	-	-	-	-	-	0.3725
Yes	61	37.9	14	30.4	23	23.7	98/106	92.5	-
No	5	3.1	0	0	3	3.1	8/106	7.5	-

MP, medical practitioners; HRSP, health and rehabilitation sciences practitioners; NP, nursing practitioners.

## Discussion

The increasing use of ChatGPT in healthcare and education reinforces the need to incorporate it into HPE. However, ChatGPT presents both opportunities and challenges, and limited data are available to guide its utilisation in local settings. Additionally, the perspectives of healthcare professionals regarding AI technology may influence its adoption within an institution.^11^ In this study, variations in the knowledge, attitudes and practices were observed among the professional groups. These findings may be interpreted within the context of the TAM, which proposes that technology adoption is influenced by users’ perceptions of its usefulness and ease of use. Although not directly measured in this study, these variations may reflect differing levels of AI literacy, utilisation and perceptions regarding its value, limitations, risks and benefits. Intergroup differences found in this study may also arise from limited interprofessional educational (IPE) activities; however, this was not assessed specifically in the study. IPE can be used to foster knowledge sharing and interprofessional communication and promote greater consistency in perspectives regarding new technology.^12^ Differences in perceptions regarding the integration of ChatGPT in healthcare training were also found in other studies.^3,13,14^

The comparatively lower proportion of nurses who had heard of ChatGPT may be because of a range of factors, including differences in age, access to learning opportunities and exposure to emerging digital technologies; however, the underlying determinants of awareness were not specifically assessed in this study. Nevertheless, this finding was consistent with other studies reporting that 20% – 40% of nurses were unaware of ChatGPT.^2,15^ In the absence of structured AI literacy programmes, unfiltered information about ChatGPT may emanate from social media, work colleagues, friends and internet sources. A recent large-scale South African survey found that most respondents became aware of AI technology through social media, with only 4% citing formal education and 2% indicating their workplaces and professional education as sources of exposure.^16^ Chat Generative Pre-Trained Transformer has a beneficial role in nursing education,^15,17,18^ and targeted activities are encouraged to leverage its potential benefits.

The small majority of respondents overall who agreed that ChatGPT improves the standard of teaching and learning, and supported its use by students, suggests that many respondents still had reservations. However, this reflects participants’ perceptions and not objectively measured educational outcomes. Medical practitioners accounted for the largest proportion, indicating their disagreement and uncertainty in this regard. In the absence of robust and convincing evidence, reservations about using ChatGPT in medical education may be associated with risk and patient safety concerns. Medical education requires accuracy, and misinformation may result in significant negative consequences for patients.^19^ In addition, students may have a limited capacity to analyse information and identify potential errors.^19^ Physicians have also expressed concerns that ChatGPT’s responses were superficial, lacked scientific evidence and required verification.^20^

However, a growing body of research highlights the potential benefits of ChatGPT, which include personalised and cost-effective learning, the creation of real-life case scenarios, eliminating patient risk in training students and almost instant feedback.^3,7,17,21,22^ Appreciating the differences of opinion in the literature, the strong agreement shown by participants overall that ChatGPT should be developed further was understandable.

Importantly, the study also highlighted that approximately two thirds of respondents expressed concerns regarding ChatGPT’s potential to generate inaccurate information, facilitate academic misconduct and negatively affect independent thinking. These concerns resonate with findings from other studies in which inaccurate information, limited knowledge, incorrect references and data bias have been reported.^3,23^ Plagiarism emerged as a dominant concern in the study, which was broadly consistent across all the groups. Similar concerns have also been highlighted in other studies, including a lack of awareness of academic misconduct and its potential legal consequences.^24,25^ Regarding the impact of ChatGPT to reduce independent thinking, some evidence suggests that while ChatGPT can support critical and independent thinking by allowing students to generate new ideas and analyse information, overuse and over-reliance may compromise independent thinking, problem-solving skills and students’ motivation for self-reflection and critical evaluation.^26^

The low utilisation of ChatGPT by the professionals in this study may be attributed to unfamiliarity, uncertainty about its benefits, apprehension about its risks and the lack of training; however, these factors represent possible explanations rather than confirmed determinants. It may also be related to the finding that less than a quarter of the respondents using ChatGPT reported receiving prior training on the application. In addition, the use of other AI technologies such as Google Bard, DeepSeek and Meta AI are alternatives to ChatGPT; however, these technologies were not assessed in this study. Nevertheless, a large majority found that ChatGPT was easily accessible within the institution and expressed a need for further training. Reflecting on the TAM, these findings suggest that improving AI literacy and training may enhance both perceived ease of use and perceived usefulness, thereby facilitating the adoption of ChatGPT in HPE. The variation in the number of responses reflects the questionnaire design, whereby questions relating to prior training, institutional accessibility and the need for further training were completed only by a subgroup of respondents who indicated that they currently used ChatGPT.

The findings from this study emphasise the importance of expanding awareness and implementing targeted and structured interprofessional training programmes that incorporate AI technology. Targeted initiatives should highlight the benefits and limitations, and include risk mitigation strategies to ensure the safe, ethical and effective use of ChatGPT in HPE. The inclusion of evidence-based data in these programmes is likely to foster more confidence among healthcare professionals about its integration in HPE. The ease of accessibility to ChatGPT and strong demand for further training are encouraging and suggest that these initiatives probably will be successful and should therefore be implemented.

To our knowledge, this is the first study in South Africa to assess the knowledge, attitudes and practices regarding ChatGPT across a diverse group of healthcare professionals in a teaching hospital setting. The teaching hospital presents a real-world environment where interprofessional and work-based training of healthcare professionals is conducted. The study’s findings provide valuable insights that contribute to the integration of AI tools such as ChatGPT into HPE.

### Limitations

A limitation of the study is the fact that it was conducted in a single teaching hospital, and the findings may not be generalisable to other teaching hospitals within the country and globally. Furthermore, the MP group consisted of hospital-based specialists and registrars. The perspectives of other medical professionals, such as laboratory-based professionals and pathologists, may differ because of variations in their educational, clinical and research responsibilities. Moreover, this was a cross-sectional point-in-time study, and future longitudinal studies should include a broader range of healthcare professionals and evaluate changes in knowledge, attitudes and practices over time as evidence regarding the use of ChatGPT in HPE continues to evolve.

## Conclusion

Chat Generative Pre-Trained Transformer has the potential to transform HPE, although it presents both opportunities and challenges. Further research and development are required, particularly in resource-limited settings to support the creation of AI-inclusive curricula. Variations in knowledge, attitudes and practices exist among health professionals, which may be attributed to differing levels of exposure, literacy, utilisation, curricular differences and work-related experience. An interprofessional approach should be emphasised within the teaching hospital setting to enhance information sharing about ChatGPT and similar AI tools. Despite concerns, enthusiasm for the further development of ChatGPT and training on its use remains strong, and targeted educational programmes are likely to be successful. Future guidelines should also incorporate ethical guardrails to ensure the safe, responsible and effective use of ChatGPT in HPE.
